# Dry eye disease in axial spondyloarthritis: heterogeneous ocular surface dysfunction involving tear film instability and tear production from the AIDA network registry

**DOI:** 10.3389/fmed.2026.1851512

**Published:** 2026-08-03

**Authors:** Valeria Caggiano, Antonio Vitale, Jessica Sbalchiero, Abdurrahman Tufan, Guillermo Arturo Guaracha-Basañez, Perla Ayumi Kawakami-Campos, Andrea Hinojosa-Azaola, Rahime Duran, Hamit Kucuk, Gaafar Ragab, Piero Ruscitti, Giuseppe Lopalco, Anastasios Karamanakos, Lampros Fotis, Mahmoud Ghanema, Paola Cipriani, Maria Morrone, Aikaterini Dimouli, Katerina Kourtesi, Samar Tharwat, Abdelhfeez Moshrif, Jurgen Sota, Adrián Mayo-Juanatey, Oksana Boyarchuk, Amr Edrees, Patrizia Barone, Francesco Carubbi, Maria Sole Chimenti, Alessandro Conforti, Maissa Thabet, Daniela Opris-Belinski, Şükran Erten, Mohamed Tharwat Hegazy, Maria Antonietta Mazzei, Chiara Piscitello, Bruno Frediani, Alberto Balistreri, Carlos Cifuentes-González, Alejandra de-la-Torre, Luca Cantarini, Claudia Fabiani

**Affiliations:** 1 Department of Medical Sciences, Surgery and Neurosciences, Research Center of Systemic Autoinflammatory Diseases and Behçet’s Disease Clinic, University of Siena, Siena, Italy; 2 European Reference Network for Rare Immunodeficiency, Autoinflammatory and Autoimmune Diseases (ERN-RITA) Center, Azienda Ospedaliero-Universitaria Senese, Siena, Italy; 3 Division of Rheumatology, Department of Internal Medicine, Gazi University Hospital, Ankara, Türkiye; 4 Department of Immunology and Rheumatology, Instituto Nacional de Ciencias Médicas y Nutrición Salvador Zubirán, Mexico City, Mexico; 5 Department of Ophthalmology, Instituto Nacional de Ciencias Médicas y Nutrición Salvador Zubirán, Mexico City, Mexico; 6 Rheumatology and Clinical Immunology Unit, Department of Internal Medicine, Faculty of Medicine, Cairo University, Giza, Egypt; 7 Rheumatology Unit, Department of Biotechnological and Applied Clinical Sciences, University of L’Aquila, L’Aquila, Italy; 8 Department of Precision and Regenerative Medicine and Ionian Area (DiMePRe-J), Policlinic Hospital, University of Bari, Bari, Italy; 9 Department of Rheumatology, Evangelismos General Hospital, Athens, Greece; 10 Department of Pediatrics, Attikon General Hospital, National and Kapodistrian University of Athens, Athens, Greece; 11 Rheumatology and Immunology Unit, Department of Internal Medicine, Mansoura University, Mansoura, Egypt; 12 Department of Internal Medicine, Faculty of Medicine, Horus University, New Damietta, Egypt; 13 Department of Rheumatology, Faculty of Medicine, Al-Azhar University, Assiut, Egypt; 14 Department of Rheumatology, Doctor Peset University Hospital, Valencia, Spain; 15 Department of Children’s Diseases and Pediatric Surgery, Ternopil Regional Children’s Hospital, I. Horbachevsky Ternopil National Medical University, Ternopil, Ukraine; 16 Division of Rheumatology, University of Missouri–Kansas City, Kansas City, MO, United States; 17 Pediatric Rheumatology Unit, Department of integrated Maternal-Child and Reproduction Activity, AOU “Policlinico-San Marco”, Catania, Italy; 18 Internal Medicine and Nephrology Division, Department of Life, Health and Environmental Sciences, ASL1 Avezzano-Sulmona-L’Aquila, San Salvatore Hospital, University of L’Aquila, L’Aquila, Italy; 19 Rheumatology, Allergology and Clinical Immunology Unit, Department of Systems Medicine, University of Rome Tor Vergata, Rome, Italy; 20 U.O. Medicina Generale, Ospedale San Paolo di Civitavecchia, ASL Roma 4, Civitavecchia, Italy; 21 Department of Internal Medicine, Farhat Hached University Hospital, Faculty of Medicine of Sousse, University of Sousse, Sousse, Tunisia; 22 Department of Internal Medicine and Rheumatology, Carol Davila University of Medicine and Pharmacy, Bucharest, Romania; 23 Department of Rheumatology, Ankara Yıldırım Beyazıt University, Ankara Bilkent City Hospital, Ankara-Tüürkiy; 24 Rheumatology Unit, Department of Internal Medicine, Jahra Hospital, Al Jahra, Kuwait; 25 Unit of Diagnostic Imaging, Department of Medical, Surgical and Neuro Sciences, University of Siena, Siena, Italy; 26 Bioengineering and Biomedical Data Science Lab, Department of Medical Biotechnologies, University of Siena, Siena, Italy; 27 Department of Ocular Immunology, Ophthalmology Research Center, SIGMA Clinic, Cali, Colombia; 28 Centre of Excellence in Ocular Inflammation, Colombian Visual Science and Translational Eye Research Institute (CERI), Bogotá, Colombia; 29 Neuroscience Research Group (NEUROS), Neurovitae Center for Neuroscience, Institute of Translational Medicine (IMT), School of Medicine and Health Sciences, Universidad del Rosario, Bogotá, Colombia; 30 Ophthalmology Unit, Department of Medicine, Surgery and Neurosciences, University of Siena, Siena, Italy

**Keywords:** dry eye disease (DED), Schirmer test, spondyloarthritis, tear break-up time (TBUT), tear film instability, tear production

## Abstract

**Objective:**

Dry eye disease (DED) has been described in axial spondyloarthritis (axSpA), but patterns of tear fluid and ocular surface involvement remain incompletely characterized. This study aims to evaluate tear film instability and tear production in axSpA and explore the heterogeneity of DED manifestations.

**Methods:**

Axial spondyloarthritis patients and healthy controls underwent non-invasive tear break-up time (niTBUT), Schirmer test, and Ocular Surface Disease Index (OSDI)- 6 evaluation. Group differences were assessed using niTBUT thresholds (<10 s and <5 s).

**Results:**

Mean niTBUT was significantly lower in axSpA patients than controls (7.85 ± 5.63 vs. 10.61 ± 5.27 s; *p* < 0.001); niTBUT <5 s, but not <10 s, differentiated groups (*p* = 0.003 and *p* = 0.105, respectively). ROC analysis identified an optimal niTBUT cut-off of 5.15 s. Schirmer values also discriminated axSpA patients from controls, with an optimal threshold of 4.5 mm. Agreement between niTBUT and Schirmer classification was weak (Cohen κ = 0.18). OSDI-6 scores were not significantly associated with pathological niTBUT. The niTBUT values were significantly associated with patients’ age, but the diagnosis of axSpA accounted for the main determinant of niTBUT <5 s (likelihood ratio test, *p* = 0.026).

**Conclusion:**

Within the present cohort, a niTBUT <5 s showed greater discriminative performance than the conventional 10-s cut-off and may identify a subgroup of axSpA patients with more severe tear film instability. Aging contributes to tear film instability, but axSpA independently contributes to severe niTBUT impairment. Tear film instability and reduced tear secretion are both involved in DED affecting axSpA patients.

## Introduction

Dry eye disease (DED) is a multifactorial disorder of the ocular surface characterized by disruption of tear film homeostasis, tear film instability, and variable degrees of inflammation and neurosensory dysfunction (1–3). The Tear Film & Ocular Surface Society (TFOS) Dry Eye Workshop (DEWS) III report has recently refined the diagnostic framework of DED, emphasizing the central role of objective measures of tear film instability and advocating for severity-based stratification rather than reliance on traditional, potentially arbitrary cut-off values (4). Within this updated conceptual framework, non-invasive tear film break-up time (niTBUT) has emerged as a key parameter for evaluating tear film stability and identifying clinically relevant alterations of the ocular surface (4, 5).

Axial spondyloarthritis (axSpA) is a chronic immune-mediated inflammatory disease characterized by axial skeletal involvement and a well-recognized spectrum of extra-articular manifestations (6, 7). Ocular involvement in axSpA is classically represented by acute anterior uveitis, which is strongly associated with human leukocyte antigen (HLA)-B27 (8–10). However, ocular surface alterations such as dry eye disease have received considerably less attention despite increasing evidence suggesting that tear film dysfunction may also occur in this population (11–13). From a pathophysiological perspective, dry eye may arise from reduced aqueous tear production, instability of the tear film, or the coexistence of both mechanisms (14, 15). In patients with axSpA, it remains unclear whether ocular surface dysfunction predominantly reflects immune-mediated alterations related to systemic inflammation, or whether it represents the cumulative effect of age-related changes, chronic inflammatory burden, and treatment exposure (12, 13). Clarifying these mechanisms is important for understanding the clinical significance of ocular surface involvement in axSpA.

In our previous study, we reported an increased prevalence of dry eye features in patients with axSpA compared with healthy controls, suggesting that ocular surface dysfunction may represent an additional component of the axSpA spectrum (13). The previous study primarily focused on establishing the association between axSpA and dry eye disease through symptom assessment, tear production tests, tear film stability measurements, and ocular surface staining. In contrast, the present analysis specifically re-examines tear film instability according to the recently published TFOS DEWS III framework, with particular emphasis on niTBUT thresholds, their discriminative performance, and the determinants of severe tear film instability. However, that analysis was conducted according to earlier diagnostic frameworks and did not specifically address how different thresholds for tear film instability might influence disease classification or the interpretation of associated clinical determinants. Considering the evolving classification of DED introduced by TFOS DEWS III, reassessing tear film stability using both conventional and updated severity-oriented criteria may provide a more accurate characterization of DED manifestations in axSpA (4).

Accordingly, the present study re-evaluates tear film instability in a well-characterized cohort of patients with axSpA using niTBUT measurements. The discriminatory performance of conventional and severity-oriented niTBUT thresholds is compared, and the independent contribution of demographic and disease-related factors to niTBUT impairment is assessed.

## Methods

### Study design and population

This prospective study accounts for a secondary analysis of a previously described cohort of patients with axSpA and healthy controls included to assess DED in such a disease (13). Both the ophthalmologists and the operator conducting the statistical analysis were blinded to the participants’ clinical status. The original study design, inclusion and exclusion criteria, and demographic data collection have been reported elsewhere (13).

A total of 71 patients fulfilling the Assessment of SpondyloArthritis International Society (ASAS) classification criteria for axSpA (6) were included in the study. Demographic and clinical information was obtained from the international AutoInflammatory Disease Alliance (AIDA) Network registry dedicated to spondyloarthritis^1^ (16). In parallel, 19 healthy controls without systemic inflammatory or ocular surface diseases were included as a comparator group. Exclusion criteria for all participants included active ocular infection, ocular surgery within the previous 6 months, contact lens use, and systemic conditions known to independently affect tear film homeostasis.

The participants enrolled were not using topical ophthalmic medications at the time of the ocular examination or during the month preceding the assessment. Both patients and controls underwent a complete ophthalmologic evaluation, including assessment of the tear film and ocular surface. Data were collected from the right eye of each enrolled participant.

All patients and healthy controls provided signed informed consent for the study, which was conducted according to the Declaration of Helsinki. The study was approved by the Ethics Committee of the Azienda Ospedaliero-Universitaria Senese, Siena, Italy in June 2019 (Ref. N. 14951).

### Ophthalmologic assessment

All participants underwent a standardized ophthalmologic evaluation, including assessment of tear film stability and tear production. In the present analysis, tear film stability was evaluated using a non-invasive, device-based TBUT measurement system based on automated optical detection of tear film disruption, without fluorescein instillation (Keratograph 5M, Oculus, Wetzlar, Germany). Measurements were recorded in seconds. To avoid inter-eye correlation bias, only the right eye was considered for statistical analysis. NiTBUT was analyzed both as a continuous variable and as a categorical variable using different diagnostic thresholds. In addition to the conventional cut-off of 10 s (4), a more stringent threshold of 5 s was applied to reflect a severity-oriented classification.

Tear production was assessed using the Schirmer test without topical anesthesia, which is adopted in the assessment of DED in systemic rheumatic diseases to evaluate both basal and reflex tear secretion (17).

A standardized Schirmer strip (I-DEW tearstrips; Tottenham Lane, London, UK) was placed over the lower eyelid margin, positioned at the junction between the middle and lateral third, for a duration of 5 min. Participants were instructed to keep their eyes closed during the procedure.

Dry eye disease was defined based on the presence of abnormal tear production, indicated by a Schirmer test value <6 mm and <10 mm after 5 min, or impaired tear film stability as assessed by niTBUT (<5 and <10 s) (4, 18).

The Ocular Surface Disease Index-6 (OSDI-6), a validated 6-item questionnaire designed to provide a rapid assessment of ocular surface symptoms, was administered to all participants to assess dry eye-related symptoms (19, 20).

### Clinical and demographic variables

Demographic and clinical data considered for analysis included sex; age at the time of ocular assessment; age at the start of ocular symptoms; age at the onset of articular manifestations (in axSpA patients); age at the time of diagnosis of axSpA; age at the start of the treatment for axSpA; axSpA disease duration; presence of psoriasis, anterior uveitis, inflammatory bowel diseases, HLA-B27 positivity; and use of medications that may influence ocular dryness, such as selective serotonin reuptake inhibitors (SSRI) or serotonin-norepinephrine reuptake inhibitors (SNRI).

### Statistical analysis

Continuous variables were expressed as mean and standard deviation (SD) or median and interquartile range (IQR), according to data distribution assessed through the Shapiro-Wilk test, while categorical variables were reported as absolute frequencies and percentages. Differences in continuous niTBUT values between axSpA patients and healthy controls were assessed using parametric or non-parametric tests as appropriate. Differences in the prevalence of pathological niTBUT according to different thresholds were evaluated using chi-square or Fisher’s exact test, according to frequency counts and expected frequencies.

Receiver operating characteristic (ROC) curve analysis was performed to evaluate the ability of continuous niTBUT and Schirmer test values to discriminate between axSpA patients and healthy controls. Optimal cut-off values were identified from ROC curves using the Youden index, defined as the maximum value of sensitivity plus specificity minus one. The area under the ROC curve (AUC) with corresponding 95% confidence intervals (95% CI) was calculated to assess the discriminative performance of each test.

To investigate determinants of tear film instability, logistic regression models were constructed using pathological niTBUT defined according to both the 10-s and 5-s thresholds as dependent variables. Odds ratios (OR) with corresponding 95% CI were calculated. Within the axSpA cohort, regression analyses were conducted to explore associations between demographic and clinical variables and pathological niTBUT.

To evaluate the independent contribution of axSpA status, pooled analyses including both patients and controls were performed. Logistic regression models were fitted with pathological niTBUT as the dependent variable and disease status included together with relevant demographic and clinical factors. Nested models were compared using likelihood ratio tests based on differences in residual deviance to determine whether inclusion of disease status significantly improved model fit.

Additional logistic regression models were performed to explore the relationship between subjective symptoms and objective tear film instability. Pathological niTBUT was used as the dependent variable, while OSDI-6 score was included either as a continuous variable. These analyses were performed both in the pooled dataset including patients and controls and in the subgroup of axSpA patients.

Linear regression analysis was performed using niTBUT as a continuous dependent variable in axSpA patients to identify factors independently associated with tear film stability. Regression coefficients (β) with corresponding 95% CI were reported.

Agreement between objective diagnostic tests for dry eye, specifically Schirmer test and niTBUT classification, was assessed using Cohen’s kappa coefficient.

All statistical analyses were performed using R software. A two-sided *P*-value <0.05 was considered statistically significant.

## Results

The present study represents a secondary analysis of a previously described axSpA cohort. A total of 71 patients with axSpA and 19 healthy controls were included; baseline demographic and clinical characteristics have been reported in detail in the original publication (13). DED symptoms were reported in 46 (64.8%) patients and 3 (15.8%) healthy controls (*p* = 0.0004).

**FIGURE 1:**
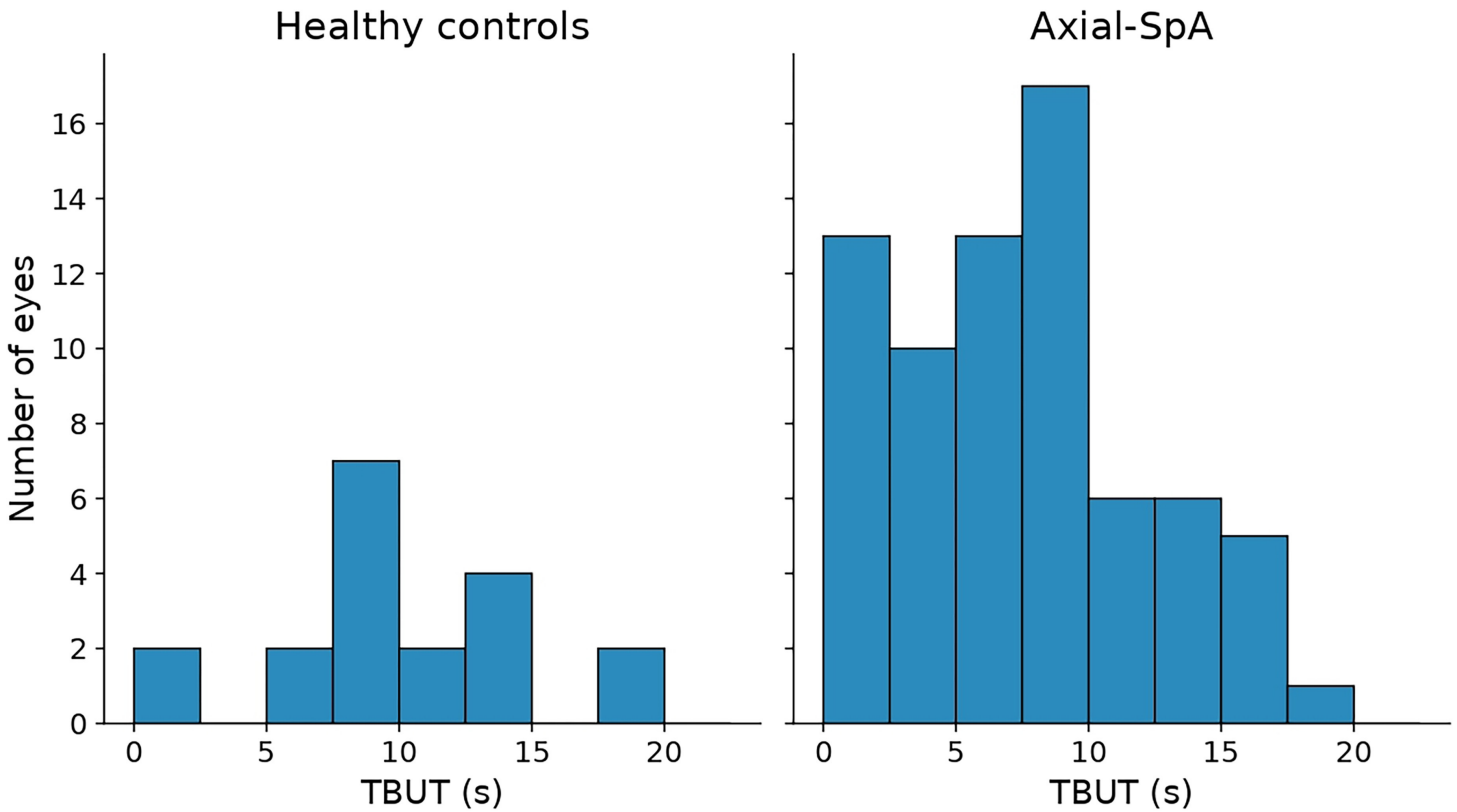
Distribution of tear break-up time (niTBUT) values in axial spondyloarthritis patients and healthy controls. Distribution of non-invasive tear break-up time (niTBUT) values in patients with axial spondyloarthritis (axSpA) and healthy controls. The figure illustrates the shift toward lower break-up times in the axSpA group, highlighting the greater burden of tear film instability compared with controls and the broader dispersion of values within the patient population.

**FIGURE 2:**
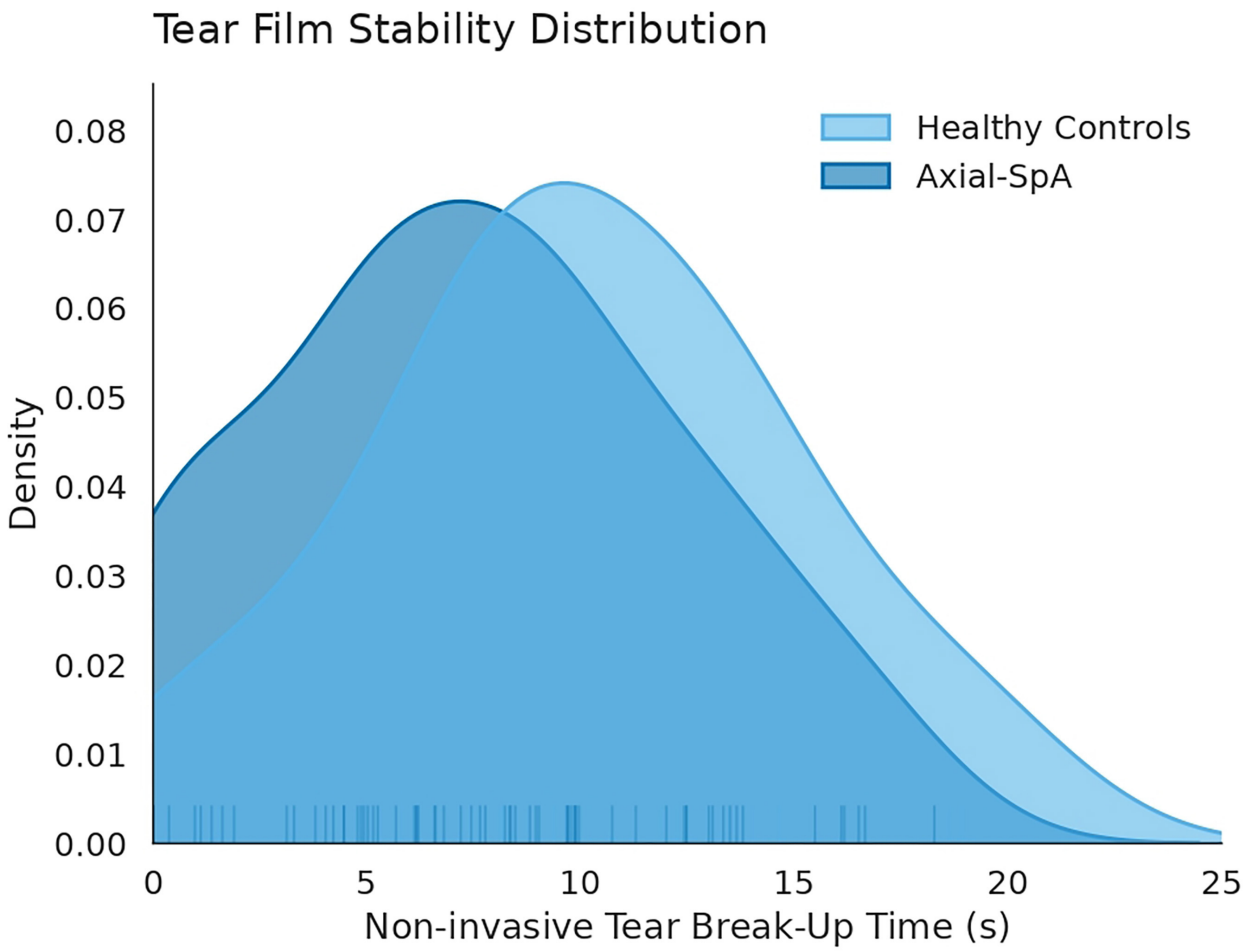
Distribution of non-invasive tear break-up time (niTBUT) in patients with axial spondyloarthritis (axSpA) and healthy controls. Kernel density curves show a leftward shift in axial spondyloarthritis (axSpA) patients, indicating reduced tear film stability compared to controls. Rug plots along the *x*-axis represent individual measurements.

Mean niTBUT was significantly lower in patients with axSpA compared with healthy controls (7.85 ± SD 5.63 s and 10.61 ± SD 5.27 s, respectively; *p* < 0.001). The distribution of niTBUT values is illustrated in Figures 1, 2.

When the conventional diagnostic threshold of niTBUT <10 s was applied, pathological niTBUT was observed in 47 of 71 (66.2%) axSpA patients and in 9 of 19 (47.4%) healthy controls (*p* = 0.105). When a threshold of niTBUT <5 s was applied, pathological niTBUT was observed in 32 of 71 (45.1%) axSpA patients and in 2 of 19 (10.5%) healthy controls (*p* = 0.003). Similarly, logistic regression analysis adjusted for age at examination showed a statistically significant association between axSpA diagnosis and severe tear film instability with niTBUT <5 s (OR: 7.56, 95% CI 1.97–50, *p* = 0.01), whereas statistical significance was lost for niTBUT <10 s (OR: 2.49, 95% CI 0.89–7.15, *p* = 0.08). Comparison of different nested logistic regression models demonstrated that inclusion of axSpA status significantly improved model fit compared with age alone to predict niTBUT <5 s (likelihood ratio test *p* = 0.026).

**FIGURE 3:**
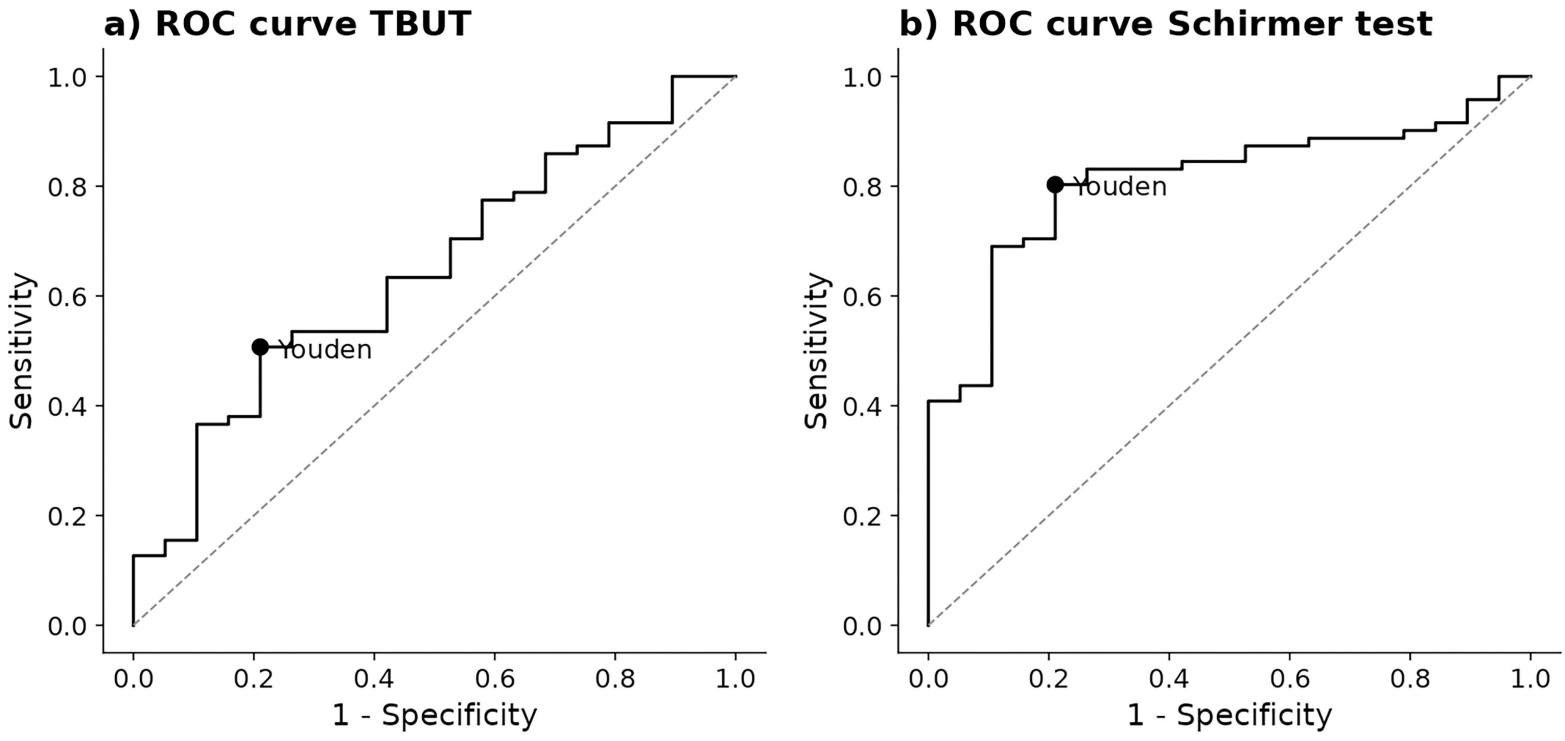
Receiver operating characteristic (ROC) curves of tear break-up time (niTBUT) and Schirmer test for discrimination between axial spondyloarthritis patients and healthy controls. Receiver operating characteristic (ROC) curves evaluating the ability of tear break-up time (niTBUT) and Schirmer test to discriminate between patients with axial spondyloarthritis (axSpA) and healthy controls. The curves illustrate the trade-off between sensitivity and specificity across different thresholds and identify the severity-oriented cut-off corresponding to the maximum Youden index, supporting the use of a more stringent threshold for optimal discrimination. In particular, the figure includes: **(a)** ROC curve for NITBUT, with an optimal cut-off value of 5.15 s; **(b)** ROC curve for the Schirmer test, with an optimal cut-off value of 4.5 mm. The Youden index points corresponding to the optimal cut-off values are indicated in each panel.

ROC curve analysis was performed to evaluate the discriminative performance of niTBUT in distinguishing axSpA patients from healthy controls. The AUC was 0.6335 (95% CI 0.508–0.759). The optimal cut-off value identified by the Youden index was 5.15 s, corresponding to a sensitivity of 48.5% and a specificity of 89.5%. ROC curve analysis based on Schirmer test values yielded an AUC of 0.77 (95% CI 0.59–0.81). The optimal cut-off value identified by the Youden index was 4.5 mm, corresponding to a sensitivity of 36.2% and a specificity of 100%. Both ROC curves are shown in Figure 3.

**FIGURE 4:**
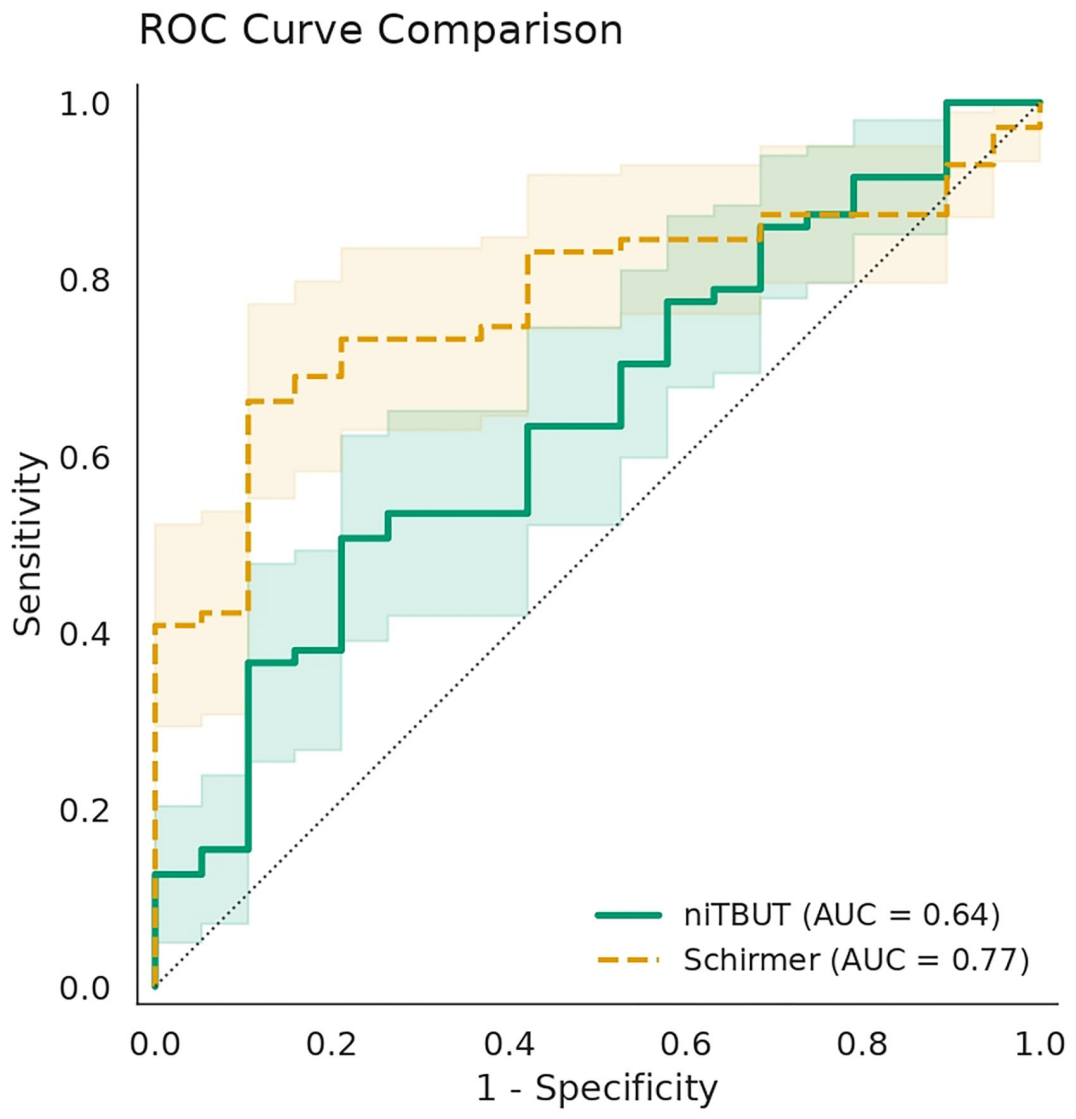
Receiver operating characteristic (ROC) curve analysis comparing non-invasive tear break-up time (niTBUT) and Schirmer test for the identification of ocular involvement in patients with axial spondyloarthritis. The non-invasive tear break-up time (niTBUT) (solid green line) showed a moderate discriminative ability [area under the Receiver operating characteristic (ROC) curve (AUC) = 0.64], whereas the Schirmer test (dashed orange line) demonstrated a higher diagnostic performance (AUC = 0.77). Shaded areas represent confidence intervals. The diagonal dotted line indicates no discriminative ability.

The comparative diagnostic performance of niTBUT and the Schirmer test is illustrated by combined ROC curves in Figure 4.

Within the axSpA cohort, logistic regression analysis was performed to investigate factors associated with pathological niTBUT defined as <10 s. Age at examination along with age at ocular symptoms onset, age at articular symptoms onset, age at axSpA diagnosis, and age at the start of treatment, and HLA-B27 positivity were associated with niTBUT <10 s. Results from univariate logistic regression models are presented in Table 1. When severe tear film instability was defined using the <5-s threshold, age remained independently associated with the outcome, while HLA-B27 positivity lost significance, as reported in Table 2.

**TABLE 1:** Univariable logistic regression analysis of factors associated with pathological tear break-up time (niTBUT) <10 s in axial spondyloarthritis patients.

Variables assessed	OR	95% CI	*P*-value
Sex	0.44	0.12–1.55	0.19
Age at the ocular examination	1.06	1.02–1.10	0.00366*
Age at articular symptoms onset	1.07	1.03–1.12	0.00168*
Age at ocular symptoms onset	1.0503607	1.01–1.09	0.0176*
Age at axSpA diagnosis	1.066495	1.03–1.12	0.00248*
Age at the start of treatment of axSpA	1.0687341	1.027–1.121	0.00225*
AxSpA disease duration	0.9829528	0.93–1.03	0.4788
Treatment duration	0.973304	0.90–1.04	0.41663
Presence of psoriasis	2.812500	0.79–13.31	0.1383
Presence of anterior uveitis	0.4146341	0.08–1.93	0.24900
Presence of intestinal involvement	0.2965116	0.05–1.47	0.1361
Presence of HLA-B27	0.1470588	0.01–0.86	0.040941*
SSRI/SSNI use	0.9722222	0.23–4.97	0.97025
Previous topical ocular treatment	1.223529	0.38–4.34	0.7398

Results of univariable logistic regression analysis evaluating demographic and clinical variables associated with tear break-up time niTBUT <10 s. Odds ratios (OR), 95% confidence intervals (95% CI), and *P*-values are provided. axSpA, axial spondyloarthritis; HLA, human leukocyte antigen; SSNI, serotonin-norepinephrine reuptake inhibitors; SSRI, selective serotonin reuptake inhibitors.

*Statistical significance.

**TABLE 2:** Univariable logistic regression analysis of factors associated with severe tear film instability (tear break-up time <5 s) in axial spondyloarthritis patients.

Variables assessed	OR	95% CI	*P*-value
Sex	1.4000000	0.41–4.87	0.587
Age at the ocular examination	1.05107798	1.01–1.09	0.00731*
Age at articular symptoms onset	1.0425785	1.009–1.080	0.0153*
Age at ocular symptoms onset	1.0483742	1.01–1.09	0.0183*
Age at axSpA diagnosis	1.048261	1.01–1.090	0.0125*
Age at the start of treatment of axSpA	1.06095857	1.02–1.11	0.00584*
AxSpA disease duration	1.0049150	0.95–1.05	0.833
Treatment duration	0.9839026	0.90–1.05	0.647
Presence of psoriasis	3.0000000	0.99–9.86	0.0571
Presence of anterior uveitis	0.6888889	0.13–3.07	0.631
Presence of intestinal involvement	0.8571429	0.15–4.21	0.848
Presence of HLA-B27	0.5476190	0.07–3.11	0.511
SSRI/SSNI use	0.3809524	0.07–1.53	0.195
Previous topical ocular treatment	1.1739130	0.39–3.49	0.771

Logistic regression model assessing determinants of severe tear break-up time (niTBUT) impairment. Odds ratios (OR), 95% confidence intervals (95% CI), and *P*-values are provided. axSpA, axial spondyloarthritis; HLA, human leukocyte antigen; SSNI, serotonin-norepinephrine reuptake inhibitors; SSRI, selective serotonin reuptake inhibitors.

*Statistical significance.

To further characterize tear film stability as a continuous functional parameter, linear regression analysis was conducted using niTBUT (seconds) as the dependent variable in axSpA patients. Age at examination was associated with niTBUT values (β = −0.17234 s per year; *p* = 0.0022). No statistically significant independent associations were observed for sex, HLA-B27 status, or psoriasis in the adjusted model. These findings are summarized in Table 3.

**TABLE 3:** Univariable linear regression analysis of determinants associated with tear break-up time (niTBUT) values in axial spondyloarthritis patients.

Variables assessed	β	95% CI	*P*-value
Sex	1.4266	−2.28 to 5.14	0.446
Age at the ocular examination	–0.17234	−0.24 to −0.066	0.00022*
Age at articular symptoms onset	−0.12654	−0.21 to −0.039	0.00511*
Age at ocular symptoms onset	−0.12544	−0.21 to −0.033	0.00864*
Age at axSpA diagnosis	−0.14740	−0.243 to −0.054	0.00244*
Age at the start of treatment of axSpA	−0.15895	−0.24 to −0.073	0.000483*
AxSpA disease duration	−0.03270	−0.17 to 0.11	0.644
Treatment duration	0.05183	−0.14 to 0.25	0.599
Presence of psoriasis	−2.6218	−5.88 to 0.64	0.113
Presence of anterior uveitis	0.8621	−3.68 to 5.41	0.706
Presence of intestinal involvement	3.3736	−1.37 to 8.12	0.1604
Presence of HLA-B27	2.7227	−2.44 to 7.89	0.295
SSRI/SSNI use	1.9939	−2.10 to 6.09	0.334
Previous topical ocular treatment	−0.4684	−3.79 to 2.85	0.7793

Linear regression model evaluating demographic and clinical variables associated with tear break-up time (niTBUT) as a continuous measure of tear film stability. Regression coefficients (β), 95% confidence intervals (95% CI), and *P*-values are provided. axSpA, axial spondyloarthritis; HLA, human leukocyte antigen; SSNI, serotonin-norepinephrine reuptake inhibitors; SSRI, selective serotonin reuptake inhibitors.

*Statistical significance.

When assessing the agreement between the Schirmer test and niTBUT in identifying DED in patients with axSpA, the agreement was weak and not statistically significant (Cohen κ = 0.18, *p* = 0.15).

In the overall cohort, OSDI-6 score analyzed as a continuous variable was not associated neither with niTBUT <10 s (OR 1.28 per 1-point increase, 95% CI 0.3–4.8, *p* = 0.72), nor with niTBUT <5 s (OR 1.61, 95% CI 0.48–5.69, *p* = 0.44).

## Discussion

In this secondary analysis of a previously characterized cohort of patients with axSpA, ocular surface involvement was examined through objective assessments of both tear film stability and tear production (13). The present findings provide further insight into the spectrum of DED manifestations in this population considering the recent TFOS DEWS III report on the diagnosis of DED (4).

Tear film instability appeared to be more pronounced in patients with axSpA compared with healthy controls. Indeed, niTBUT values were significantly lower in patients than in controls. When attempting to identify a cut-off capable of discriminating between the two groups, the conventional 10-s threshold, commonly used to identify patients with DED (1, 5), proved inadequate for distinguishing axSpA patients from controls. In contrast, a 5-s cut-off appeared to be more appropriate. Supporting this observation, ROC curve analysis identified an optimal niTBUT threshold of 5.15 s for discriminating axSpA patients from controls. Although this threshold was associated with a relatively low sensitivity, its high specificity suggests that more severe tear film instability may represent the ocular surface alteration most strongly associated with axSpA, while reducing the likelihood of misclassifying subjects with borderline or age-related tear film changes. This finding is noteworthy, as it closely approximates the commonly used 5-s threshold for severe tear film instability. The convergence between the ROC-derived value and this lower threshold suggests that marked reductions in niTBUT may represent the most clinically relevant alteration of tear film stability in this population, consistent with the TFOS DEWS framework, which also includes cut-off values consistent with more severe disease in terms of tear film instability (1, 4, 5, 21).

Alterations of the ocular surface in axSpA did not appear to be limited to tear film instability alone. When tear production was evaluated using the Schirmer test, ROC analysis demonstrated a moderate ability to discriminate axSpA patients from healthy controls, with an optimal threshold of 4.5 mm. This finding is consistent with previous evidence indicating that aqueous-deficient and evaporative mechanisms frequently coexist in dry eye disease (11, 14, 15, 22). In support of this interpretation, no significant agreement was observed between the Schirmer test and niTBUT according to Cohen’s kappa coefficient. This lack of concordance suggests the presence of different DED subtypes, which may be captured by distinct diagnostic methods assessing different aspects of the disease. Therefore, in this patient population, the combined assessment of the Schirmer test and niTBUT may be useful to better characterize both tear film instability and aqueous tear secretion impairment in line with different types of ocular surface involvement and a multifactorial nature of DED (11).

It should also be noted that the Schirmer test without use of topical anesthesia may retain relevance in rheumatologic settings, as both basal and reflex tear secretion may be assessed in Sjögren’s disease and other immune-mediated rheumatic diseases, including axSpA, where evaluation of tear production may provide additional insight into ocular surface involvement (11).

This may also explain the lack of association between subjective symptoms, as assessed by the OSDI-6, and objective measures of tear film instability. This finding is consistent with the well-recognized dissociation between symptoms and clinical signs frequently reported in dry eye disease. Several mechanisms, including ocular surface inflammation, neurosensory alterations, and interindividual variability in symptom perception, may contribute to this discrepancy (5, 23–25).

Consistent with the well-documented decline in tear film stability observed with physiological aging, likely reflecting structural and functional changes of the lacrimal functional unit and ocular surface homeostasis, age emerged as an important determinant of tear film stability in the present analysis (14, 26). For this reason, age at ocular assessment was included as a covariate in regression models. Despite this adjustment, the association between disease status and niTBUT remained significant. Furthermore, comparing two regression models for niTBUT, one including age alone as a predictor and the other adding disease status, it was observed that inclusion of the diagnosis significantly improved the predictive performance of the model. This confirms that, independently of age, having a diagnosis of axSpA significantly identifies patients with niTBUT <5 s. These findings suggest that systemic disease may contribute to ocular surface alterations beyond the effects of physiological aging (12, 13).

Overall, the findings from the present study highlight the importance of a comprehensive ophthalmologic assessment in patients with axSpA. A multidimensional evaluation of the ocular surface may be necessary, as different diagnostic tests capture distinct components of tear film dysfunction (5).

Several limitations should be acknowledged. The relatively small sample size may limit the detection of more subtle associations. NiTBUT and Schirmer test were assessed in a single eye to avoid inter-eye correlation bias, which may not fully capture bilateral variability. The relatively small number of healthy controls may have affected the precision of ROC-derived estimates and regression analyses. Therefore, the identified niTBUT threshold should be considered exploratory and requires validation in larger independent cohorts before any potential clinical application. In addition, information regarding systemic therapies was incomplete for a substantial proportion of patients, preventing a reliable treatment-stratified analysis. Therefore, we could not assess whether conventional immunosuppressive agents, biologic therapies, or Janus kinase inhibitors may have influenced the observed ocular surface findings. Nevertheless, systemic therapies may potentially influence ocular surface inflammation and tear film stability through modulation of inflammatory pathways involved in dry eye disease (22). Finally, the cross-sectional design precludes conclusions regarding temporal relationships between systemic disease activity and ocular surface alterations.

In conclusion, ocular surface involvement in axSpA encompasses both tear film instability and reduced tear production. This underscores the need for a comprehensive assessment of ocular surface function in such patients, which should not only include the evaluation of tear film instability and ocular surface damage, but also tear production.

## Data Availability

The raw data supporting the conclusions of this article will be made available by the authors, without undue reservation.

## Footnotes

1
https://aidanetwork.org/en/register/spondyloarthritis
